# Transactions of the Medical Society of London. New Series. Vol. I

**Published:** 1846-04

**Authors:** 


### Transactions of the Medical Society of London. New Series. Vol I. 8vo. pp. 221. London, 1846. Highley.

The London Medical Society was established in the year 1773; it is the oldest of any in England. In 1788, the first volume of its Memoirs was published, the opening paper being written by Dr. Lettsom on the character of Esculapius.

This series, amounting to six volumes, was continued until the year 1805. The first part of the seventh volume was not published until 1810, and the second part not before 1817. Things had therefore began to flag for some time before the late very lengthened suspension. From the last-mentioned date, the Society ceased to exist as a publishing body; its voice was not heard throughout the land, save only by an occasional notice of its proceedings in one or other of the weekly periodicals.

The Council has now very wisely determined to imitate the example of the founders of the Society, by bringing under public notice a selection of the papers that have been read during the last two or three years. We hail the appearance of the present volume as an earnest of increased exertion, and cordially join in the hope expressed by the Council, that "the renewal of these annals will prove a fresh stimulus to the zeal of the Fellows to furnish, from time to time, materials for a series of volumes, calculated to strengthen the bond of scientific union, and thus promote the advancement of medical knowledge, and the consequent alleviation of human suffering."

I. This volume commences with a well-written and very interesting paper by Mr. Thomas Bell, Professor of Zoology in the King's College, and author of several works of acknowledged repute upon this department of Science. It is entitled "Observations on the use of the Microscope, in investigations connected with Anatomy, Physiology, and Pathology," and formed the substance of the annual oration delivered before the Society, in March 1844.

The author takes a rapid review of the most important discoveries that have been achieved in microscopical anatomy from the time of Malpighi in about the year 1660 down to the present period. He dwells with appropriate emphasis upon the astonishing merits of this celebrated man, pointing out the great extent and variety of his researches, and shewing how nearly he anticipated many of the most vaunted discoveries of modern times, notwithstanding the very imperfect nature of the instruments which he used.

Scarcely inferior to Malpighi, was his contemporary Leuwenhoeck, a native of Delft in Holland. "His range of investigation was boundless; scarcely a structure to which he could gain access, eluded his active and untiring eye; and the Philosophical Transactions of our Royal Society teemed for a considerable period with the results of his indefatigable labours." The names of Grew and Lieberkuhn next come in for notice: to the latter we are indebted for that beautiful appendage to the microscope, the silver cup (which still bears his name), by which opaque objects can be examined with concentrated reflected light. A lamentable hiatus now occurs in the annals of microscopic research. Hewson, our countryman, has the merit of again restoring the microscope to its proper place in anatomical and physiological pursuits. But his example was not followed as it deserved. Passing over "the coarse and incorrect observations of Sir Everard Home," our author brings us down to the present age. He pays a well-merited compliment to the "busy London Merchant" (Mr. Lister), to whom the scientific world is so much indebted for the discovery of the true principle of achromatism, and the consequent great improvement that has taken place in the construction of our microscopes. The wonderful disclosures, that have been achieved in the unravelling of minute structures during the last 15 or 20 years, must be known to every one. Mr. Bell, while he admits the great interest and even the importance of many of these discoveries, is not blind to the evils of nimble observation and unadvised generalization that have set in, like a strong current, into the field of physiological enquiry. "It will be very useful," says he, with excellent judgement, "if a degree of philosophical scepticism—a disposition of mind as wholesome in the pursuits of science, as it is baleful in the consideration of questions of a more solemn nature—shall interpose to prevent or modify the hasty and baseless conclusions, to which the love of distinction and fame, or the enthusiasm of a sanguine temper and an undisciplined mind, is so apt to lead."

In our notice of the subsequent articles in these Transactions, we shall arrange them not as they stand in the volume, but as they may present some point of relationship to, or may best serve to illustrate, each other.

### II. Observations on the Cause and Treatment of Stammering, &c. By *John Bishop*, F.R.S. &c.

Mr. Bishop commences his paper by alluding with surprise to the opinions of some recent writers, that "Stammering is the result of some abnormal condition of one or more of the organs which compose the vocal apparatus, such as the frænum linguæ, the velum, uvula, tonsils, or some of the muscles of the tongue," and by reprobating the "many surgical mutilations of these organs which have taken place both at home and abroad." He then very properly remarks that "a little attention to the vocal apparatus during the act of stammering will, however, soon enable a competent observer to discover that the affection is entirely of a functional character, and not dependent on any organic lesion of the parts concerned in the production of articulate language."

That any man of common sense should have allowed himself to adopt the absurd notion of Dieffenbach that the infirmity in question depends on a malformation of some of the muscles of the tongue, or the still more silly idea of Yearsley that enlargement of the tonsils is the offending cause, has always surprised, nay grieved, us not a little; for the circumstance is discreditable to the profession. In the following passage, Mr. Bishop succinctly explains the physiology of Stammering.

"It has been truly remarked by Sir Charles Bell that the consent of a great number of organs is necessary for the production of the most simple sound, or the articulation of a single word. Dr. Arnott also observes that command over the organs of speech is acquired in the same manner as over all the muscular organs of the body; as for example, in walking, skating, fencing, and performing on musical instruments. Agreeably to this view, it will be found, on analysis, that want of synchronous action of each part of the vocal mechanism concerned in speech is essentially and fundamentally the cause of stammering. The most common species of stuttering is an attempt to articulate before the vocal ligaments are brought into vibratory action, and the breath vocalized; and, therefore, in order to remove this kind of imperfection, we have nothing more to do than to instruct the patient in the art and method of using the vocal apparatus properly. To effect this purpose, it is necessary to call upon the patient to vocalize the breath so as to utter a continuous sound, as by singing a note in music. This he should first do without making any attempt to articulate a syllable; and then, on repeating the same sound, should endeavour to articulate the word required. This he will be enabled to do immediately if, during the whole time that the attempt is made to pronounce the articulate sounds, care be taken that the glottis is kept in action by the vocalization of the air issuing from the lungs." P. 27.

By following this simple plan, the worst stammerer may be made to utter whole sentences without impediment, in the course of a few minutes; and, by perseverance, a cure may very generally be quickly effected.

The interruption of vocalised breath—the real cause, be it remembered, of Stammering—may arise from several different causes, and be *seated* in different parts of the vocal apparatus. It may be caused by closure either of the valve of the glottis, or of the isthmus of the fauces, or of the lips themselves; or, again, it may be owing to the tip of the tongue being turned up so as to come in contact with the palate. To ascertain which of these four conditions is present in any particular case, the following directions are given.

"When the glottis is involuntarily closed during speech, the articulation of all the vocal sounds is affected. The closing of the isthmus of the fauces necessarily affects, not only those letters and syllables where it ought to remain open, but its irregular action will also cause an involuntary movement, on attempting to pronounce the words beginning with the gutturals *k*, *g*, &c. The involuntary or misdirected action of the tip of the tongue affects the dentals and palatals, such as *l*, *n*, *t*, and therefore words containing or beginning with those letters. That of the lips not only affects the labials, but may likewise impair the pronunciation of all the other letters. The ear of any experienced person may easily detect, without ocular inspection, whether it is the valve of the glottis, or the aperture of the isthmus of the fauces, which is instrumental in producing the interruption, by observing the manner in which the breath is suppressed, and suffered to escape. The same observation will enable him to discriminate the effect produced, when the sounding column of air is stopped by the tip of the tongue. When the lips are the cause of the interruption, we have ocular demonstration to assist us in distinguishing the organs which are instrumental in causing the defect. The mode of relief, however, for all these kinds of stammering is nearly the same, the principle above proposed applying to them all." P. 29.

The remarks of Mr. Bishop on the subject of (functional) Aphonia will not detain us long. The loss of power in most instances is referred by him to relaxation of the fauces and soft parts adjacent to the larynx, or to loss of elasticity in the membranes of the vocal tube. By far the most efficient remedy is the application, with a camel's-hair brush, of a solution of lunar caustic—ten grains to an ounce of water. It will be necessary to administer tonic medicines at the same time, if the constitution is weak.

The influence of a damp atmosphere on the quality of the voice is sometimes remarkable. "When Grassini, the celebrated singer, came from Italy to England the humidity and variable temperature of the air in this country, so relaxed the membranous portions of her respiratory tube, that her voice sank nearly an octave in pitch, and was changed from a soprano to a contralto character. The effect however was, that she acquired great celebrity in consequence of the fine quality and range of her lower notes; but on returning to the milder climate of Italy, the soprano range of voice returned, and she lost at the same time the power of uttering that range of notes which had conferred on her voice its attractive character. In this case the relaxed condition of the vocal membranes was productive of beneficial effects, but it is presumed that such cases are extremely rare."

When Aphonia arises from a purely nervous cause, as from a sudden fright, all remedies, however skilfully devised, will often fail. Time and cheerfulness of spirits are then the best remedies.

### III. A Case of Cynanche Laryngea in which Laryngotomy was performed. By *H. P. Robarts*, Esq.

A gentleman, 58 years of age, had been for several days suffering from general feverishness, slight difficulty of swallowing, and soreness of the right tonsil, when, rather suddenly one evening, the dysphagia increased so much that he could not get anything down his throat. He spoke with pain and considerable effort; but his voice was loud and not hoarse or altered in character. There was great tenderness about the left side of the throat. Although there was no dyspnœa, the patient could not "snuff-up" with his nostrils—a symptom said by Dr. M. Hall to be diagnostic of laryngeal disease. Leeches and then fomentations were applied; an active purge of calomel and jalap administered, and the inhalation of the vapour of hot vinegar and water ordered. The symptoms became aggravated; the breathing was considerably distressed at times, and attended with slight sibilation; and there were occasional fits of coughing. A blister was applied to the left side of the neck, and Belladonna ointment rubbed in over the right. "It was proposed also," says Mr. R. "to administer belladonna internally; but the pain and difficulty of deglutition defeated this part of the plan."* The difficulty of breathing became very much increased; so much so, indeed, that there were all the signs of impending asphyxia: this was in the afternoon of the following day on which the

* We cannot perceive what benefit could have been expected from such a remedy. How was it that an emetic was not administered in the early stage of this case, and the nauseating effect kept up afterwards by frequently-repeated small doses of antimony or ipecacuan? In all acute laryngeal affections, this is a most important part of the medical treatment; steady perseverance in it will often supersede all other remedies.

active symptoms had commenced. Laryngotomy was accordingly performed without delay, to the immediate relief of the patient. The surface, that had been vesicated, was dressed with mercurial ointment; and this was likewise rubbed into the arm-pits: one grain of calomel was also ordered to be given every hour. The temperature of the room was kept up to 70°. Next day, the patient was much better; he breathed quietly through the tube in the larynx, so long as it remained free from mucus; whenever it was at all obstructed, his breathing was much distressed. The use of the tube was, after a day or so, discontinued; and gradually the patient began to breathe with comfort even when the wound in the larynx was plugged up.

The calomel was continued, until ptyalism was induced. In the course of a week or two, the patient was entirely restored to health.

Mr. Robarts, in his remarks appended to this case, alludes to the extreme rapidity with which laryngitis sometimes proves fatal—doubtless by inducing a spasmodic contraction, in addition to the inflammatory swelling, of the glottis. The alarming symptoms are not to be attempted to be combated by inordinate depletion, as seems to have been the case in the last illness of Washington; "his physicians, from an excess of zeal, and from an over-anxiety to save so illustrious a character, abstracted 90 ozs. of blood in little more than 12 hours;"—and this too in an old man upwards of eighty years of age!

### IV. A Case of Obscure Abdominal Tumor. By *W. C. Dendy*, Esq.

A gentleman, 31 years of age, while recovering from an attack of Influenza, perceived one evening (Oct. 16th,) that there was a considerable fullness on the left side of the abdomen; it seems to have come on very suddenly. It increased so rapidly that, in the course of 16 hours, it reached from the margin of the ribs to Poupart's ligament, extending across the umbilicus, projecting into the loins, and resting, as it were, on the crista of the ileum. It presented a uniform shape, and felt soft and doughy; the right side of the abdomen was more or less distinctly sonorous on percussion. No inconvenience was experienced except from a certain degree of dyspnœa, induced no doubt by the pushing up of the diaphragm. The urine was highly charged with the lithates. Two days subsequently, the patient became affected with frequent paroxysms of most severe neuralgia in the track of the lumbar plexus. Messrs. Dendy and Pilcher attributed the symptoms to some intestinal cause—"probably," says the former of these gentlemen, "not fæcal impaction, but an accumulation of morbid secretion, and that rather *iliac* than colic." It deserves to be noticed that, on two or three former occasions in early life, the patient had been somewhat similarly affected with a swelling on the left side of the abdomen; the symptoms had then speedily yielded to the use of aperients. On the present occasion, as well as on that which preceded it four years before, the attack seemed to have followed the use of large doses of the nitrate of potash.*

* On the penultimate attack, we gather that there was a long-continued torpor.

21st. We read that "the symptoms continued the same; the neuralgia was very frequent, often very severe; *the tumour occasionally altering, but soon resuming its former shape*." The excretions from the bowels were very scanty and unhealthy.

The neuralgia extended to the sacral and sciatic nerves; the patient lost strength, and became emaciated; scarcely any feculent evacuation could be procured. On one occasion, it is reported, that "on auscultating a fold of intestine at the edge of the tumour, a metallic gurgling is heard, as if air and water were washing about a distended tube." We also learn that, after the administration of a copious enema, the abdominal tumefaction, and the neuralgic pains were greatly aggravated. On a subsequent date, it is stated that the swelling was "seemingly intestinal."

We are now told for the first time that "Dr. Bright believes the kidney or its capsule to be the chief seat of disease." On the following day we read: "no alvine evacuation; stupor recurs regularly at 3 p.m. Three distinct elastic nodules perceived on the tumour, bearing the superficial character of cysts." A week after this date, the first somewhat solid motion was passed; and, on the same day, the urine was found to be "turbid throughout, depositing evidently globules of pus, forming about a moiety of the secretion. They mix with the urine on being shaken, but there is no solution; heat produces little change; *liquor potassæ* coagulates the pus in firm long strings." From this time it would seem that the abdominal swelling gradually subsided. The purulent admixture with the urine continued for several days, and then gradually became less and less. In the course of two or three weeks from this period, the patient's health was completely re-established.

Mr. Dendy favours us with a rather tedious examination of the various morbid conditions that *might have been* the cause of the symptoms in such a case as the preceding. Two very acute pathologists, he tells us, regarded the swelling as of splenic origin; while another of his scientific friends actually suspected it to be caused by the development of a malignant (we suppose encephaloid) growth. The presence of a serous or hydatidic cyst was also thought of. Allusion is made by Mr. Dendy to two cases in which an obscure abdominal tumour was of this nature: in one of them, after a month of extreme suffering, masses of hydatids passed *per anum*, and the swelling disappeared.

It is not easy to discover what opinion Mr. D. really thinks the most probable: if he leans to any, it is to that which regarded the kidney or its capsule as the seat of the disease. He remarks that—

"Dr. Bright, who has deeply studied these cases, has observed that the kidney often approaches the left hypochondrium, and looks like spleen: but if we trace it back towards the loins, we find its chief bulk further back and more fixed, so that if the patient rests on his hands and knees, the renal tumour does

---

of the bowels, and also "that, about three weeks from its commencement, very copious albuminous deposit was observed in the urine, and this was followed by gradual subsidence of the tumour—an event strongly confirming Dr. Bright's opinion. (Mr. Dendy has rather oddly omitted to tell us, as yet, what this was). It is right, however, to state that the diminution was also preceded by a severe and perilous hypercatharsis and collapse, the result of drastic purgatives."

not fall forward equally with the splenic, and on percussion the intestine is proved to be between the former tumour and integuments,—not so in the latter case. In my own case percussion did not confirm this latter sign, and indeed the passing of the tube behind a portion of tumour would entice the mind towards the spleen, the intestines rarely, I believe, passing *before* a splenic engorgement. If the kidney be hypertrophied to excess, the error of diagnosis will be extremely probable. For instance, in the case related Dr. Thompson of Perth, in which the kidney weighed seven pounds imperial, and in which the symptoms were nearly parallel with those of my own, there was but one opinion, I believe, as to its seat,—that it was in the spleen. With all this perplexity, if the graphic pages of the very excellent pathologist to whom I have alluded be carefully studied, the diagnostic error can seldom arise." P. 83.

Allusion is made to three cases recorded by Dr. Johnson in the *Medico-Chirurgical Review* for 1823: in these, the capsule of the kidney formed an immense cyst or bag, which gave rise to great tumefaction outwardly.

The perusal of Mr. Dendy's case has certainly led us to the opinion that not a few of the symptoms were due to intestinal distension, arising from an atonic or semi-paralytic condition of the affected part, coupled with a general torpid state of the liver, and other chylopoietic viscera. The repeated application of sinapisms, and the internal use of the fætid gums, along with bitter purgatives, and perhaps occasional mercurials, would have answered better, we suspect, than anodynes, croton oil, enemata of hot water, &c. The whole catalogue of the symptoms—the urinary excepted—reminds us of what we have witnessed, more than once, in some hysterical females. This view of the case is a good deal strenghtened by the history of that very curious and interesting case of abdominal tumour recorded in the number of this Journal for October, 1840, and which occasioned so great discrepancy of diagnostic opinion among some of the leading medical men in Paris. That the peccant cause, in that instance, was in part of a hysterical character, cannot reasonably, we think, be doubted. There are some interesting points of resemblance between it and the present case of Mr. Dendy's.

### V. Remarks on Hydatids. Illustrated by a Case of Hydatid Abscess of thirty years' duration. By *Theophilus Thompson,* M.D., F.R.S., &c.

The prefatory observations need not detain us, especially after our ample notice of Klencke's work on the subject in the last number of this Journal. With but one short extract, we shall proceed to give a summary of the interesting case related by the learned President of the Society.

"Various causes have been assigned for the development of hydatids, such as moist situations, and particular kinds of diet. In the lower animals, the influence of food in this respect is considerable. Rabbits, for example, when fed on green vegetables are much more liable to hydatids than when fed on bran; but, in the human subject, I have not been able to trace any especial influence of food or climate in their production; except that the free use of salt seems unfavourable to their development, and that sailors enjoy a remarkable immunity from these intruders. Judging from the facts which have come to my knowledge, the most frequent cause of their appearance in man is mechanical injury; and it is reasonable to suppose that a blow, by impairing the vitality of a suffering organ, may lessen its ability to resist the development of any ova which it may contain."* P. 164.

At the close of the year 1821, after giving birth to a son, the patient became affected with a swelling at the umbilicus, which was regarded as a hernia; a truss was accordingly applied.† It would seem that the tumour remained pretty stationary for nearly eleven years, (her health meanwhile had been very ailing), when at length it burst, and discharged a quantity of offensive matter, in which there were hundreds of cysts, varying from the size of a grape to that of a melon. (?) The opening healed in five months; but, during the six months subsequent to its closure, the tumour re-appeared; and then a second swelling, to the right of the umbilicus, made its appearance: this also, in course of time, broke and "discharged the same sort of stuff as the former." The patient lived for about six years after this, and eventually died in the beginning of 1842.

*Dissection.*—"A swelling protruded from the umbilicus, three inches in diameter, less firm than fatty tumour, less yielding than abscess; there was a somewhat larger tumour inclining a little to the right side. On opening the former tumour, a quantity of fluid resembling a mixture of chalk and water, having a faint offensive odour, escaped, intermingled with numerous small masses of a substance resembling thick honey. On pressing the second tumour, it was obvious that similar matter escaped from it into the first, and both these receptacles were manifestly supplied from some other reservoir. An incision made from one of the swellings through the abdominal muscles passed upwards, on the right side, into a capacious cyst, or rather conduit, in form resembling intestine; greyish in its interior, and surrounded with circular fibres resembling muscle, but thicker than those of intestine. This pipe was in contact with the peritoneum, lying between that membrane and the abdominal muscles. The intestine immediately behind was uninflamed, and lay loosely in the enclosing peritoneum. The conduit above described, which was about two inches in diameter, passed upwards for about six inches, where it terminated in a pouch, which seemed to have had an opening in its upper part obliterated by a pale, puckered, contracted cicatrix, surrounded with small nodules. This pouch adhered above to the peritoneum, and was connected by coagulable lymph to the under surface of the liver, where was a cavity, three inches in diameter, containing pus of a natural character, intermixed with fragments of hydatid cysts. Between the lobes of the liver was a white firm tumour of the size of a small pear, full of hydatid cysts, about an inch or less in diameter, having fibrous yellowish layers externally, and thin membranous cysts within. Some weeks before death this tumour had

* "Strong confirmation of this opinion has lately been furnished by Klencke, who observed acephalocysts in cows' milk, and the ovules swimming in the serum of milk; and injected fluid, charged with these ovules, into the veins of different animals. It is an interesting fact that in various instances, a few weeks after the experiment, he found acephalocysts largely developed in those organs on which he had inflicted mechanical injury."

† Previous to this period, and shortly after her first confinement, she had received a kick on the abdomen; and in consequence, as she believed, of the blow, became affected with a moveable tumour in the epigastrium, and under the ribs on the left side.

been felt, and yielded a crepitating sensation, like that produced by peritoneal adhesions. The gall-bladder, three times its natural size, contained some bile, but was distended with compressed hydatid cysts. About eight small firm hydatids were imbedded in the liver, near the surface of this organ, which was dark-coloured and friable. The mesentery was studded with numerous cysts of a similar character. The peritoneum above the umbilicus, for an extent of several inches, was much reddened, thickened, and adherent to the bowels." P. 167.

### VI. Case of Obstructed Bowel, terminating fatally. By *C. Waller*, M.D., &c.

An exceedingly corpulent, middle-aged, man, who had been for many years the subject of an enormous irreducible scrotal hernia, was rather suddenly seized with a violent pain in the abdomen; the pain was not constant, but recurred at intervals; and there was no feverish excitement of the system. On the previous day (28th Nov.), the bowels had acted very freely from two purging pills taken the night before. All the symptoms of obstruction of the bowels now set in; obstinate constipation in spite of powerful purgatives, vomiting, and great distention. As the herniary swelling however remained free from any tenderness, the seat of the mischief was not supposed to exist there. On the 30th, he was bled and had a warm bath; and the use of aperients with enemata was steadily persevered in. Mr. Solly visited him, along with Dr. Waller, on the following day; and both agreed that, as the hernia was free from tenderness and tension, an operation was scarcely justifiable. Leeches were however applied upon the swelling, and small doses of calomel and opium given at regular intervals. The pulse hitherto had not been at all altered from the standard of health. On Dec. 2nd, as there was no amendment, Dr. W. ordered a drastic purgative, as an *ultima ratio*. Accordingly, five grains of calomel and ten of gamboge, and as many of scammony, were administered in one dose; it was retained on the stomach, although hitherto the vomiting had been unappeasable. Next day, the patient was in every respect worse; the bowels had not acted, and the feeling of distension most agonising. It was accordingly determined to give the patient the chance of relief from an operation. It is thus described :—

"The incision was made in the usual manner, and on opening the sac, a large portion of omentum, loaded with fat, protruded itself. This mass was adherent in many parts to the sac. Behind this there was a large portion of colon, healthy in its appearance. The adhesions of the omentum were partly cut way, and partly torn through, and when freed, a ligature was put round it, and a portion cut away, which was afterwards found to weigh twelve ounces and a half. Another large portion of colon was then discovered at the posterior part of the sac, of an equally healthy appearance. Mr. Solly, finding considerable difficulty in returning these portions of the gut, passed his finger up to the neck of the sac, and, with a hernia knife, divided a few of the external fibres forming the margin of the abdominal ring: he next drew down successively both portions of intestine, to ascertain *whether* and *where* they had been strictured, but there was not the slightest appearance of injury on any part of the bowel, which was then returned into the cavity of the abdomen: this part of the operation was effected with difficulty, in consequence of its great distention. The edges of the wound were then united by sutures, over which gum plaster was applied, and upon this a strong compress of lint, the whole being secured by a T bandage." P. 134.

The patient died early on the following morning.

*Dissection.*—"No traces of disease manifested themselves." The herniary sac was quite empty, and exhibited not a trace of inflammation. All the intestines, small and large, were enormously distended with flatus; one portion of the transverse colon was not so much distended as the rest, but it was not at all contracted; nor did it present any appearance of having been strictured, although it was this part of the large gut that had been contained in the sac. There was a *very large accumulation of fluid fæces* both in the large and in the small intestines.

Dr. Waller, upon reviewing the history of the case, comes to the conclusion (and we think rightly), that "the greater (?) portion of the intestinal tube was in a state of paralysis," or paralytic atony. He professes himself to be not friendly to the use of drastic purgatives, but prefers mild laxatives, in most cases of obstructed bowels: we cordially join with him in this respect; we have often seen much mischief produced by the indiscreet use of violent medicines in such cases; and even in Dr. Waller's own case, it will be observed, the symptoms become decidedly aggravated after the drastic powder was administered, even although there was actually no mechanical obstruction in the intestinal canal. Dr. W. regrets now that he did not add a small portion of Strychnia to the laxatives: it is very questionable whether it would have been of the least use. A warm spirituous purgative—an ounce, for example, of tincture of rhubarb, or castor-oil with brandy—would have been much better. No mention is made of any outward applications (the leeches excepted) to the abdomen. In all cases of obstructed bowels, the continued application of flannels wrung out of boiling water, so hot as to prove a decided and even a painful rubefacient, is a most important adjuvant to the use of internal remedies.

With respect to the use of the knife, it may indeed be proper to have recourse to "an exploratory operation," when there is obstruction of the bowels in connexion with an existing hernia, although the local symptoms of strangulation be entirely absent; but surely nothing could justify such rude handling of the contents of a hernial sac, as seems to have been practised in the present case, not to mention the very unnecessary removal of a portion of omentum that weighed no less than 12 ounces and a half! That the death of the patient was accelerated by the operation cannot, we should think, be doubted. Dr. Waller seems to have regretted its performance; for he rests its justification on the always questionable, and often most pernicious, old saying "*anceps remedium melius quam nullum.*"

VII. There is a "Case of Internal Strangulation," related in a subsequent part of these Transactions. The patient had been long subject to attacks of severe abdominal pain, accompanied with partial obstruction of the bowels; but hitherto they had always yielded to the use of purgative medicines. At length, upon one occasion, the symptoms were more obstinate; and although Mr. Bryant says, that "he had but little doubt of the existence of important inward mechanical obstruction in the intestinal canal," we find him ordering two purgative pills to be taken every four hours! The patient died upon the third day; and here is the report of the state of the bowels that was found upon dissection.

"On opening the cavity of the belly, a process of omentum was observed to be, as it were, drawn down, having a fixed point of attachment. On examining this more carefully, it was found that this cord-like extension of the omentum was attached to a mass of mesenteric glands, situated behind the duodenum and ileum, from which mass a very *thin membranous cord* was observed, presenting the appearance of a portion of the mesentery, drawing down a finger-like process of the jejunum, which embraces itself, as it were, in common with the ileum, about 3 inches before the termination of that part of the bowel in the cæcum, giving rise to perfect obstruction. The stomach and duodenum were found more than ordinarily capacious, but not distended, containing only about a pint of dark coloured fluid.

"On examining the mesentery, there was evidences of the same tendency, at several points, to draw down one side of the intestine, forming small diverticula. The convolutions of the small intestines were slightly agglutinated by the recent effusion of adhesive matter, and the surface of the serous membrane bearing all the characters of inflammation." P. 146.

The more that we consider these cases of intestinal obstruction, and compare them with each other, the more we are convinced that, on the whole, it would be much better for patients if not an atom of purgative medicine was administered, at least by the mouth; and if the treatment was invariably limited to the use of outward applications, of enemata, and, if need be, of leeches and the lancet.

### VIII. A Case of Ulceration of the Stomach and Duodenum.—By *W. C. Dendy*, Esq.

A lady, 29 years of age, of a delicate and (suspected) tubercular constitution, had an attack of Hæmatemesis in the Summer of 1844. In May, 1845, after enjoying the festivities of a christening party, she was seized about 11 p.m. with vomiting and diarrhœa: her dinner, we are told, consisted of salmon and lobster-sauce, fruit, and port-wine—a bad mess certainly for an ailing person. Next morning, when Mr. Dendy visited her, she was evidently *in extremis*, and she died an hour afterwards. The abdomen had been, for some hours before, extremely swollen, tense, and tender on pressure. Mr. D. diagnosticated ulcerated rupture of some part of the intestinal canal.

*Dissection.*—About a quart of thick reddish fluid was found in the cavity of the abdomen. Three inches from the cardia, in the lesser curve of the stomach, there was an irregular opening of about an inch long; and, close to this opening, was a band that almost contracted the stomach into two indistinct cavities. There was a second lacerated opening in the duodenum, about two inches from the pylorus: this one was nearly half an inch in length. Below the rupture, the bowel appeared to be a good deal contracted.

These cases of perforation or ulcerative rupture of the stomach or intestines are very melancholy; for they occur unexpectedly, and, as a matter of course, they defy all remedial means. Persons, who may have been quietly conversing with their friends, or siting at work, are, without any warning, suddenly seized with an indescribable sensation of pain and collapse, and die within 12 or 24 hours after the seizure. When the rupture takes place in the stomach, it is almost invariably *in its smaller curvature.*

### IX. A Case of Chronic Enteritis, with Perforation of the Intestines. By *E. H. Linnecar*, Esq.

A gentleman, 46 years of age, addicted to drinking freely of spirituous liquors, had been for two years subject to attacks of severe pain in the abdomen, accompanied with vomiting and purging. In these attacks, no symptom had ever suggested the suspicion of organic disease being present. One day, while in his ordinary health, he was suddenly seized with an acute pain in the abdomen, causing him to scream out violently: this state was speedily followed by collapse, and the patient, in the course of eleven hours from the date of the seizure, died.

"The *post-mortem* examination disclosed evidence of severe recent peritonitis, and extensive chronic disorganization. There were six or seven portions of the lower part of the jejunum, and upper part of the ilium, so constricted as scarcely to admit the passage of an ordinary sized writing quill. The intestine between the constricted parts being so much enlarged as to resemble the colon in size. On slitting open the diseased portions, the contracted parts were found in a state of ulceration, the mucous coat being entirely destroyed; and in one spot perforation had occurred, through which a small quantity of liquid feculent matter had escaped, the contents of the intestines were of a dark slate colour. The other viscera were quite healthy, with the exception of the liver, which, although apparently healthy in structure, was stained of a deep melanotic colour, to the extent of an inch within its substance; probably the effect of the gas thrown out from the intestine. The heart and lungs were perfectly healthy." P. 182.

The late Mr. Somes, M. P. died from a very similar attack, if we recollect aright. He was suddenly seized in the House of Commons with a violent pain in the abdomen, and did not survive above a day or so. The perforation was found in the duodenum.

Mr. Linnecar alludes to the rigid spasm of the abdominal muscles as a characteristic symptom of this alarming accident.

### X. On Purified Animal Charcoal, as an Antidote to all Vegetable and some Animal Poisons. By *Alfred Garrod*, M.D.

Charcoal (animal and vegetable) has the property not only of absorbing colouring matters, gases, &c., but also of throwing down certain substances from their solution in water, as iodine, lime, &c., and of removing bitter and other matters from vegetable infusions.

Bertrand in 1811 tried *wood* charcoal as an antidote to various mineral poisons; but, in the quantities used by him, it must have been perfectly inert; and Orfila has completely disproved his experiments. Dr. Garrod is not aware of any one having tried the effects of *animal* charcoal before himself. Having first ascertained that this substance* has the power of precipitating strychnia, morphia, and various other substances from a solution of hydrochloric acid, of the strength of the gastric juice, he proceeded to test its effects upon living animals.

"Two guinea-pigs were taken (about the same size); to the first, half a grain of strychnia was administered, dissolved in water, by means of a few drops of hydrochloric acid; in about five minutes the animal became tetanic, and soon died from asphixia, induced by spasm of the respiratory muscles.

"To the second animal was given the same quantity of strychnia, with the addition of animal charcoal to the solution until the bitterness was removed,—not the slightest tetanic symptom appeared.

"This last experiment was repeated several times on the animal, and always with the same result. Strychnia was then given to several rabbits, and it was found that from ¼ gr. to ⅛ gr. was sufficient to cause death; but when from ¼ gr. to ¾ gr. was administered with the animal charcoal: no injurious effects were produced, even when the animals took six times the amount of poison sufficient to destroy them." P. 197.

Dr. G. points out the necessity of using a sufficient quantity of the charcoal to neutralise the effect of the entire amount of the poison that is given. Half an ounce was found to be necessary to counteract the effects of one grain of strychnia in cats and dogs: if less were given, the animals exhibited distinct marks of poisoning.†

"Nux vomica was then given to dogs in doses of from ten to thirty grains: it was found to produce violent tetanic symptoms, or to destroy life, according to the size and age of the animal. When, however, the animal charcoal was given, in quantities varying from half an ounce to two ounces, no effect ensued. In these experiments, the antidote was sometimes given with the nux vomica,—sometimes at periods from five to fifteen minutes after, and the action of the poison was prevented: when the period was further prolonged, the poison produced some symptoms, but still life was preserved. The dogs, in the experiments, were always hungry, and their power of digestion appeared to be very much quicker, and more energetic, than that of the human subject; so that in man, the antidote would act long after the period above mentioned.

"Opium was then used as the poison, in doses of ten grains, it usually destroyed the life of the dog,—in smaller doses, it produced great stupor. Animal charcoal was found to act as a perfect antidote either when given with the poison, or before the narcotic symptoms appeared." P. 199.

Morphia and the salts of that alkaloid, when given with the antidote, were also found to be perfectly inert. The same may be said of Ipecacuan, Elaterium, Belladonna, and various other vegetable poisons, when the charcoal was administered at the same time in sufficient quantities. Even Prussic acid was not an exception; for, when 30 minims (of Scheele's strength) were given along with about half an ounce of the charcoal, no effect was produced; although another dog was quickly killed by the same dose

---

* The substance here meant is the *carbo animalis purificatus* of the London Pharmacopœia. Vegetable charcoal possesses but a feeble antidotal power: lamp-black has none.

† Half an ounce or so would seem to be required for each grain of morphia, strychnia, or any other alkaloid: the same quantity suffices for a scruple of the Nux Vomica.

of the poison, when uncombined. Perhaps the case will appear still stronger, if we look to the action of that most virulent destroyer of life, the active principle of Aconite:

"Two drachms of Morson's tincture of aconite (which causes tingling and numbness, when applied to any part) were given to a dog with about half an ounce of animal charcoal; the dog experienced no ill effect.

"Full half a grain of Morson's 'aconitina' was given to a middle sized dog; it soon caused violent vomiting and retching, which continued for some time; then, perfect loss of sensation of the whole surface came on, the heart's action became slow and feeble, and death took place. Even one fiftieth part of a grain was found sufficient to produce the same symptoms, and cause death.

"Three-quarters of a grain were then given to a dog, with about half an ounce of the charcoal, and not the slightest effect was produced, although enough was taken to destroy at least forty dogs. When aconite root, or the leaves, are given, the antidote may be administered some time after the poison." P. 200.

Dr. Garrod has also tried the effects of his antidote upon mineral poisons. Four and five grains of Arsenious acid were given to dogs, with 2 and $2\frac{1}{2}$ ozs. of the charcoal; the animals were but little affected; upon analysing the carbonaceous stools, the presence of the acid was readily discoverable. On the whole, Dr. G. concludes that "animal charcoal is equal to any known antidote of arsenic. It has greater power of removing arsenic from its solution than the hydrated sesquioxyde of iron."

The antidotal powers of the charcoal against corrosive Sublimate are not so decided, nor so much to be depended upon:

"Whites of eggs, or albumen, in any other form, would be much superior, and would not be required in such large quantities. Animal charcoal will also act as an antidote to the copper and lead salts, and to various other metallic preparations; but when an antidote is known to any of these, as to the lead salts, which is capable of forming insoluble and inert compounds with them, they would be decidedly preferable to animal charcoal." P. 202.

This paper by Dr. Garrod is by far the most original one in these Transactions; it is altogether a very valuable contribution to toxicological pharmacy, and will no doubt attract the notice of the profession generally.

XI. A Case of Extensive Asthenic Furuncular Eruption. By *J. Risdon Bennett,* M.D. Assistant Physician to St. Thomas's Hospital.

A poor child, two years and a half old, had been ailing for three or four months, when an anthrax-like swelling made its appearance on the back, between the lower angles of the scapulæ. On its most prominent part were several irregular circular openings, exposing the cellular tissue in a sloughing state. A free crucial incision was made through the centre of the tumour, and poppy fomentations and poultices were then applied. As there was a good deal of febrile excitement, purgatives and low diet were ordered. The inflammation and sloughing extended, so that on the 6th of June (eight days from the first report), "the integuments over the whole back were implicated by the disease, and there was great heat and redness. The number and peculiar character of the ulcers gave the child's back the appearance of one large honeycomb. The ulcers were preceded by small circumscribed pustules, falling rather under the denomination of psydracious than phlyzacious, being scarcely elevated above the adjacent skin, and having but little surrounding hardness or inflammation. The centre of these pustules, ere they were fully formed, broke, fell out, and left a deep excavation, presenting a sloughy base, and having a well-defined and for the most part perfectly circular margin. The holes thus made through the integuments had precisely the appearance of having been cut out with a punch, and presented little or no surrounding redness or swelling. In some instances the margin of the ulcer became undermined, and sloughed away, thus increasing the size and destroying the regularity of the original opening.

"The ulcers frequently remained stationary for a day or two, with a bright red base (after the slough had separated), and then healed. In other instances they extended in depth, and laid bare the muscles, leaving, when healed, a depressed cicatrix. When several openings occurred close together, the integument intervening between the different perforations in some instances gave way, and thus, by the union of these, one large irregular ulcer was formed. But in scarcely a single instance did the original ulcer increase by ordinary progressive ulceration. The vitality of the whole dermoid tissues appeared rather to be simultaneously and rapidly destroyed."

The use of the poppy fomentations was continued,* and quinine, ammonia, beef-tea, &c. were administered. As the quinine was imagined to cause irritation of the bowels, arsenic in small doses was substituted, upon the recommendation of Mr. Kingdon. Dover's powder, along with Hyd. c. cretâ, was given at bed-time. The local affection became gradually more and more favourable; the ulcers healed; and at length the back was entirely cicatrised. "The child, however, had, at this time, confirmed tubercular disease of the lungs, as well as mesenteric disease." It died in the following November, and the dissection amply confirmed the accuracy of Dr. Bennet's diagnosis:

"On proceeding to examine the body, the back was found to be scarred from top to bottom, and the emaciation extreme. The lungs were tubercular throughout, and tied down by firm adhesions to the pleura costalis. A large vomica existed in the upper part of the right lung, and several smaller ones in the left. There was some serous effusion in the peritoneal cavity. The liver was adherent to the peritoneum, and contained a few softened tubercles. A single crude tubercle existed in the spleen. The intestines were matted together by extensive adhesions, and the mesenteric glands were one mass of tubercular disease. Tubercular depositions existed also between the mucous and submucous coats of the small intestines; and in many spots the mucous membrane was ulcerated, and the muscular coat exposed." P. 119.

It is certainly very remarkable that nature could put forth so much reparative energy as must have been required for the cicatrisation of such an extensive ulcerated surface, when so great an amount of organic disease existed in the viscera at the time.

---

* The yeast or beer-ground poultice is generally an excellent application in such cases.

Dr. Bennett reminds the reader that the peculiar form of asthenic furuncular inflammation, illustrated by the present case, and characterised " by the round and regular form of the succeeding ulcer," was first accurately distinguished and described by M. Guersent in the 1st. Vol of the Archives Generales de Medicine, and by Dr. Copland, nearly about the same time. The French writer has thrown out the suggestion that (as there is a close structural analogy between the skin and the mucous membrane of the alimentary canal) the spontaneous perforations of the œsophagus and stomach, which are not preceded either by ulceration or gelatinous softening, may be produced in the same way as the perforations of the dermis in atonic furunculi.

XII. The case of "Lupus of the Face," narrated by Mr. Stedman, is but a sad record of the utter inefficacy of all remedies, external and internal, for the cure of the malignant form of this dreadful disease. The poor sufferer, a lady 25 years of age, lived for nearly 12 years from its first appearance, as " a small tubercular induration on the left side of the nose," which at length burst and discharged an offensive matter. Mr. Scott of Bromley at once pronounced the disease to be of a malignant character: he repeatedly applied pure nitric acid to the part, and at length an apparently perfect cicatrisation was effected. For two years, the patient thought that she had obtained a cure; but then, alas! " the old cicatrix began to lose its integrity, new tubercular indurations sprang up around it, and a sanious discharge oozed from the wound." Again, decided, but temporary, amendment was obtained from the application of the nitric acid.

Upon the recrudescence of the disease, Mr. John Scott of the London Hospital recommended the internal and external use of Iodine: the iodide of mercury was assiduously applied to the wound for two years, but without any decided benefit. The chloride of Zinc also was found to be equally ineffectual. Sir A. Cooper now was consulted; he applied pure oxide of arsenic with a camel's hair pencil to the ulcerated surface, passing it into the exposed lacrymal sac and duct. The agony produced was dreadful; and, although the application was repeated in the course of the fortnight, and with equally distressing consequences to the poor sufferer, no advantage was obtained: the disease seemed to have advanced. It may be worthy of notice that " she was always grateful to Mr. Callaway for suggesting the use of an application composed of lime-water, mucilage, and laudanum, which alleviated her agony, and afforded her comfort."

Want of space prevents our noticing the remaining articles in these Transactions.

There is a very able and elaborate paper " On the Nervous System," by Mr. Pilcher; an eloquent one " On the Incubation of Insanity," by Dr. F. Winslow; the report of a " Case of Ovarian Tumor interrupting Parturition," by Mr. Headland, and of a " Large Polypus of the Uterus during Parturition," by Mr. Crisp; and two or three others of minor consequence. There is one also by Dr. M. Hall, " On the Prevention and Treatment of Apoplexy and Hemiplegia;" but as this is little more than a mere reprint of a paper in his Observations, published last year, it scarcely deserved a place in the present volume.

